# Aging and ischemic stroke

**DOI:** 10.18632/aging.101931

**Published:** 2019-05-01

**Authors:** Mohammed Yousufuddin, Nathan Young

**Affiliations:** 1 Mayo Clinic Health System, Internal Medicine, Austin, MN 55912, USA; 2 Division of Neurology, Mayo Clinic, Rochester, MN 55905, USA

**Keywords:** aging, stroke, age-related disease

Of 795,000 strokes occurring annually in the United States, 87% are classified as ischemic strokes [1]. Aging is the most robust non-modifiable risk factor for incident stroke, which doubles every 10 years after age 55 years. Approximately three-quarters of all strokes occur in persons aged ≥65 years. As the number of people aged ≥ 65 years is projected to grow, the number of incident strokes in older adults is expected to rise, presenting major challenges for clinicians and policy makers in the foreseeable future.

**Aging and cerebral vasculature.** The complex network of the adult brain vasculature measures approximately 370 miles, receives about 20% of total cardiac output, and exchanges 20% of total blood glucose and oxygen. With aging, both cerebral micro- and macro-circulations undergo structural and functional alterations. Age-related microcirculatory changes are presumably mediated by endothelial dysfunction and impaired cerebral autoregulation and neurovascular coupling. Whereas endothelial dysfunction promotes neuro-inflammation, impaired cerebral autoregulation may lead to microvascular injury, and impaired neurovascular coupling fosters a decline in cortical function, all potential targets for future therapeutic interventions. Aging, in otherwise healthy individuals, is associated with numerous noticeable changes in human intracranial and extracranial cerebral arteries that predict the risk of future stroke.

**Figure 1:**
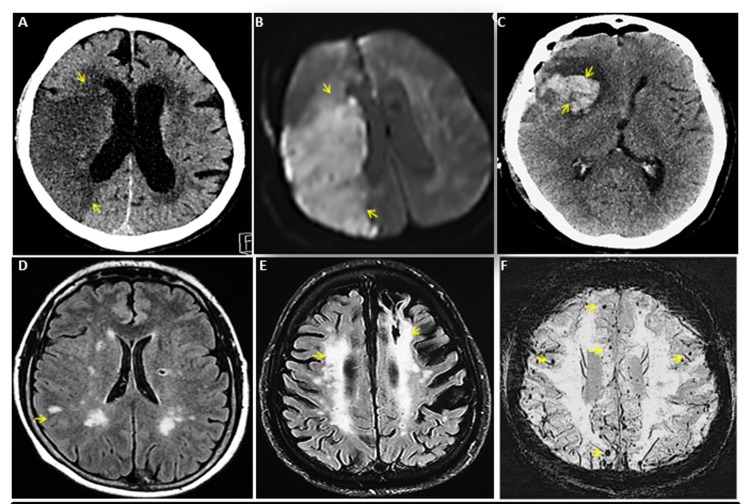
**Spectrum of cerebrovascular diseases.** (**A**) CT head without contrast of a 74 year old man with right middle cerebral artery (MCA) ischemic stroke. (**B**) Re-demonstration of acute right MCA ischemic stroke by MR brain of panel A patient. (**C**) CT head without contrast of a 75 year old woman with acute right frontal intracerebral hemorrhage with surrounding edema. (**D**) MR brain of a 54 year old woman with multiple chronic lacunar infarcts. (**E**) MR brain of an 80 year old man with white matter hyper-intensity representing severe microvascular disease. (**F**) A 73 year old woman with numerous cerebral microbleeds, few of these were shown with arrows. Source of images: These are original CT and MR images of patients collected by Mayo Clinic academics for education purpose and do not contain any patient identifier and material that has not been submitted or published elsewhere.

**Aging and silent cerebrovascular disease.** Silent cerebrovascular disease represents structural abnormalities, presumed vascular etiology, on neuroimaging not supported by clinically recognized stroke symptoms. The prevalence of silent cerebrovascular disease increases with advancing age and is recognizable as the following parenchymal lesions: 1) Silent infarcts (silent strokes), prevalence 6% and 28%, exceeds symptomatic stroke by a ratio of 10:1, 2) white matter hyperintensity or hypodensity on neuroimaging represent microvascular disease occurring in 20% to 94% older adults, and 3) cerebral microbleeds indicate silent intracerebral hemorrhages in 38% of general population aged >80 years. These conditions are age dependent and forecast increased risk of future symptomatic strokes (Figure 1).

**Aging and stroke risk factors.** A worldwide population-based study identified 10 risk factors that collectively account for 88% of the risk of stroke in all ages, which suggest that stroke is largely a preventable condition [2]. A number of additional studies recognized other important risk factors and predisposing conditions for the development of stroke. The prevalence of certain stroke risk factors including diabetes, hypertension, atrial fibrillation, and coronary and peripheral artery disease steadily increases with age. The risk factors are not equivalent in predicting the stroke risk across all age groups. The relative risks of stroke conferred by body mass index, high-density lipoprotein cholesterol, systolic blood pressure, blood glucose, or cigarette smoking declines with increasing age. Nevertheless risk factors often cluster among older adults, thereby, significantly modifying the occurrence of stroke. Emerging evidence indicates that hypertension, diabetes, and obesity may cause structural and functional alterations in the brain beyond their effect on incident stroke [3]. Despite abundant research focused on risk factors for incident stroke, the data on subsequent clinical outcomes after incident stroke are limited and even contradictory [4].

**Aging and comorbidities.** Multi-morbidity defined as the presence of two or more chronic conditions is a norm in older people and highly prevalent in persons with incident stroke, estimated at 89% for those aged ≥ 65 years and 60% for those aged < 65 years [5]. Survivors of stroke may accrue additional comorbidities during their life-time, a hypothesis that needs evaluation in a prospective study. These comorbidities, single or combined, potentially interact with conventional cardiovascular risk factor(s) to modify the risk of stroke. Comorbidities significantly influence subsequent hospital readmission, functional recovery, and mortality [5].

**Aging and therapeutic implications.** Unlike in younger adults where the evidence for primary and secondary stroke prevention is well established, and supported by robust randomized clinical trial data, the evidence base is less clear in older adults especially those aged ≥ 75 years. The benefits of primary prevention with antiplatelet, statin, or antihypertensive therapy to reduce the risk of incident stroke require an estimated life-expectancy longer than the time to benefit. National guidelines provide a grade C (selected patients) recommendation for aspirin use in adults aged 60 to 69 years and do not recommend its use in those aged ≥ 70 years, or statin therapy for people aged ≥ 75 years for primary prevention of cardiovascular events [6,7]. However, practice guidelines for the early management of ischemic stroke do not differentiate young and old except that persons aged ≥ 80 years are excluded from thrombolytic treatment within 3 – 4.5 hours of symptom onset [8]. However, subtle differences do exist in the long-term management including moderate-intensity statin for individuals aged ≥ 75 years, target blood pressure <150/90 for people aged ≥ 60 years, and less intensive glucose control allowing hemoglobin A1c levels 7%-8% in older diabetics after a stroke. It may be reasonable to incorporate information about comorbidity into clinical decision making among stroke patients with multi-morbidity to achieve optimal clinical outcome.

**Stroke mortality.** Stroke remains the 5^th^ leading cause of death, one of every 19 death, in the United States, despite decreased incidence rates (1987 to 2011) and age-adjusted death rates (2005 to 2015) [1]. Mortality associated with stroke increases with age. While mortality rates from stroke in the entire population are projected to remain stable during the next 10 years, it likely to increase in people aged ≥ 65 years [1].

**Conclusion.** To continue the gains achieved over decades, in reducing the overall incidence and mortality associated with stroke, it is important to remain focused on not only the risk factors but also on comorbidity.
